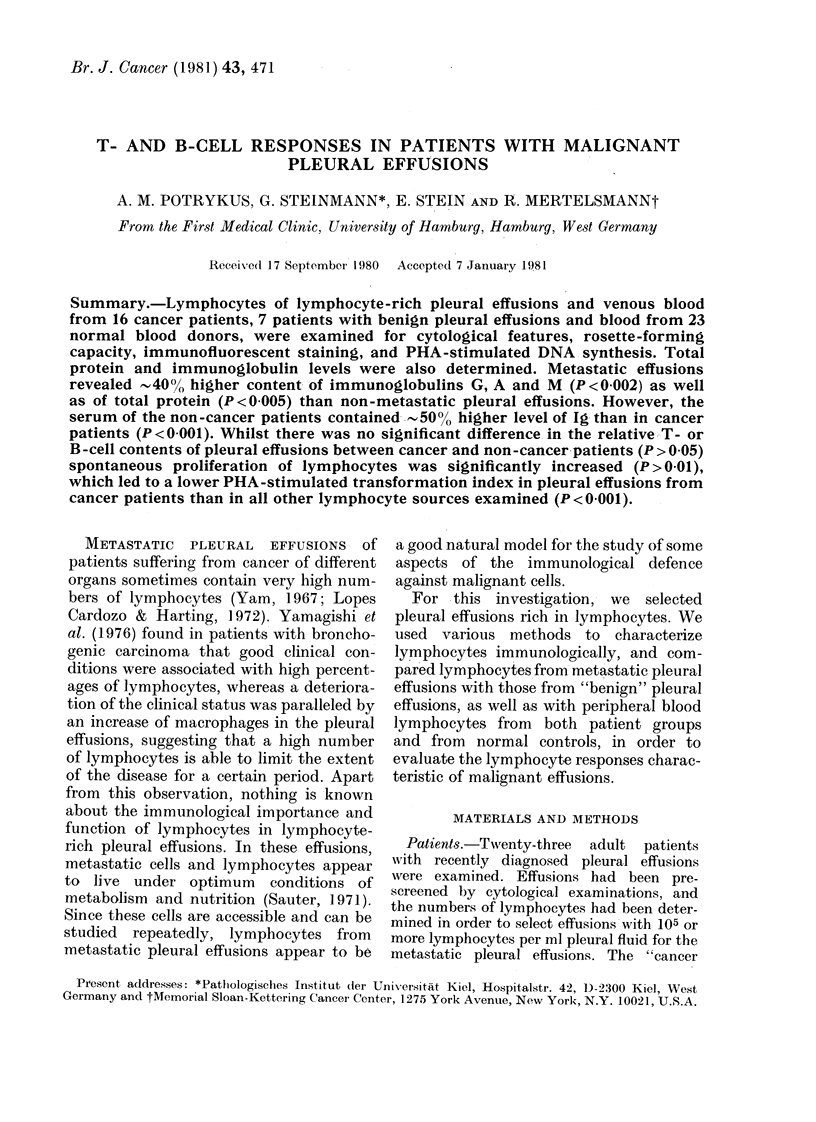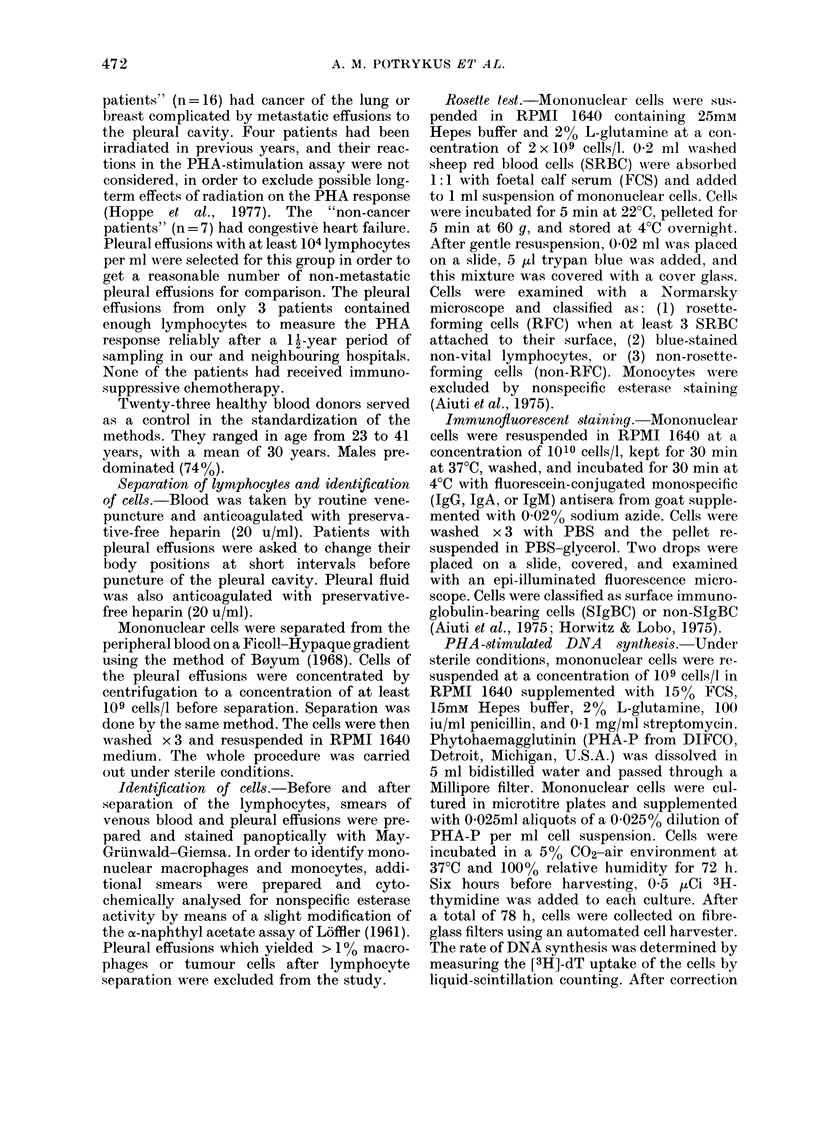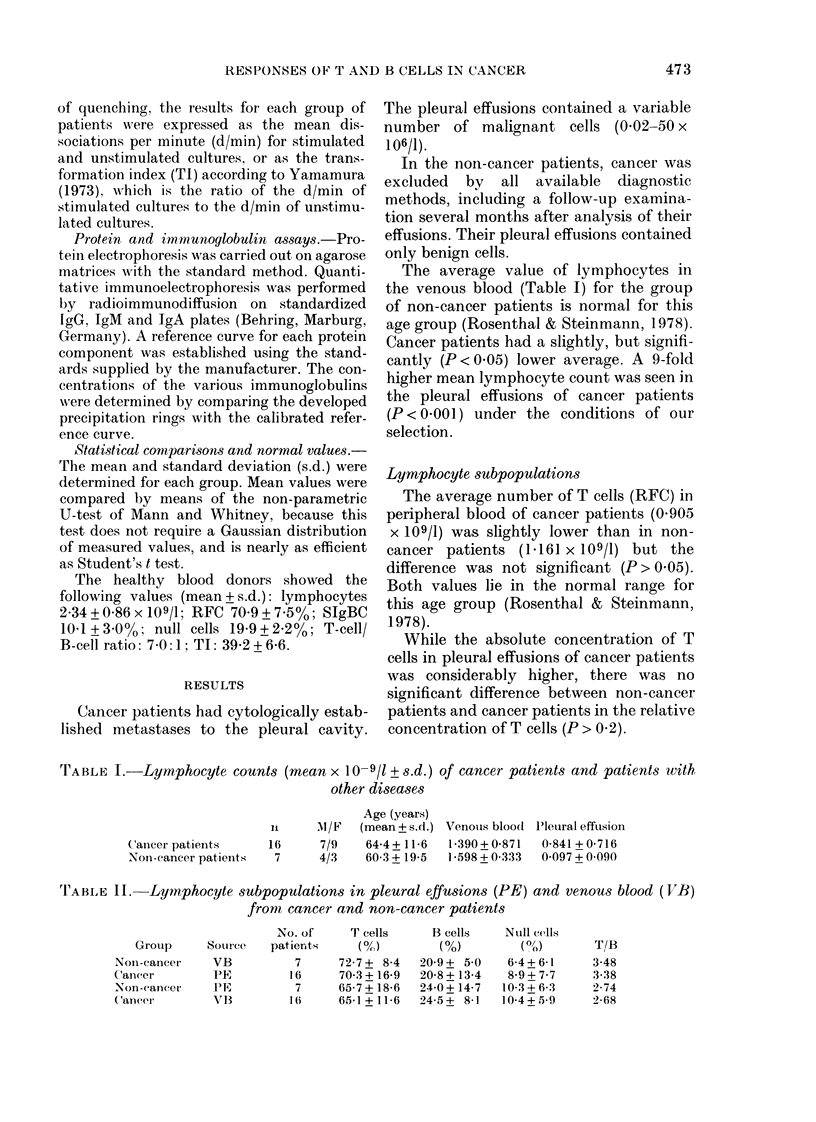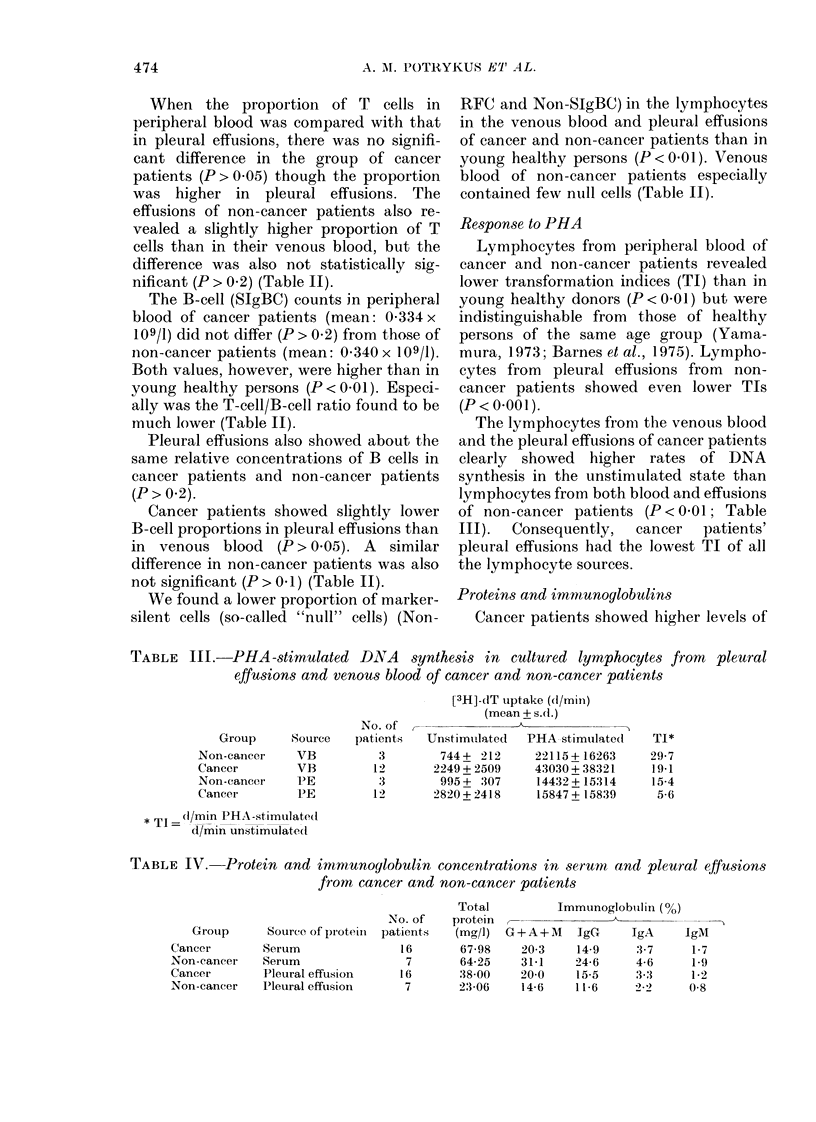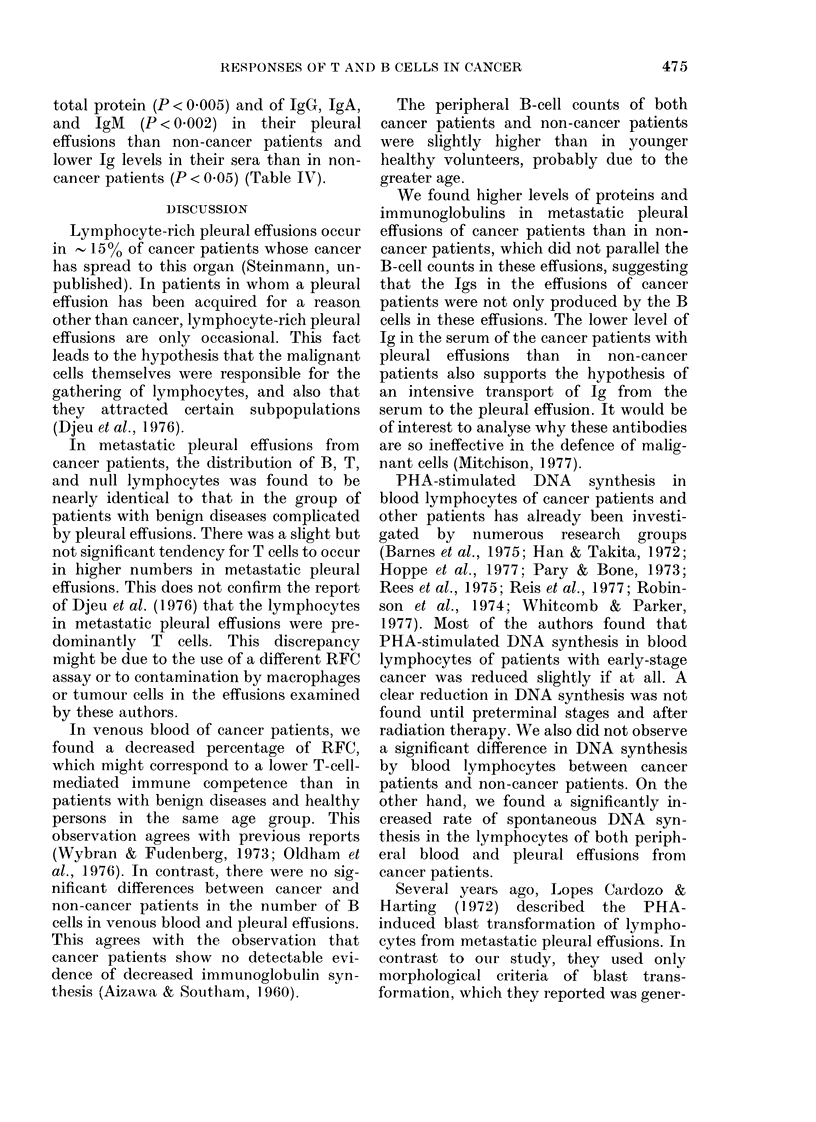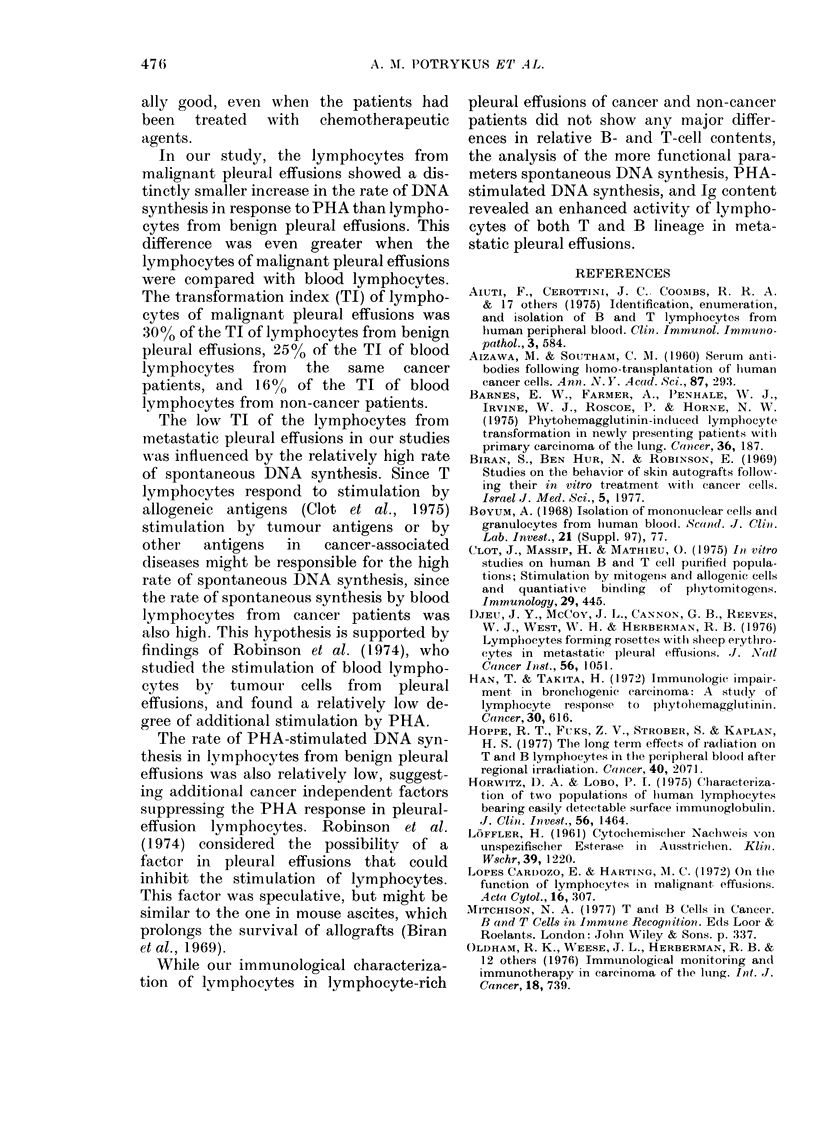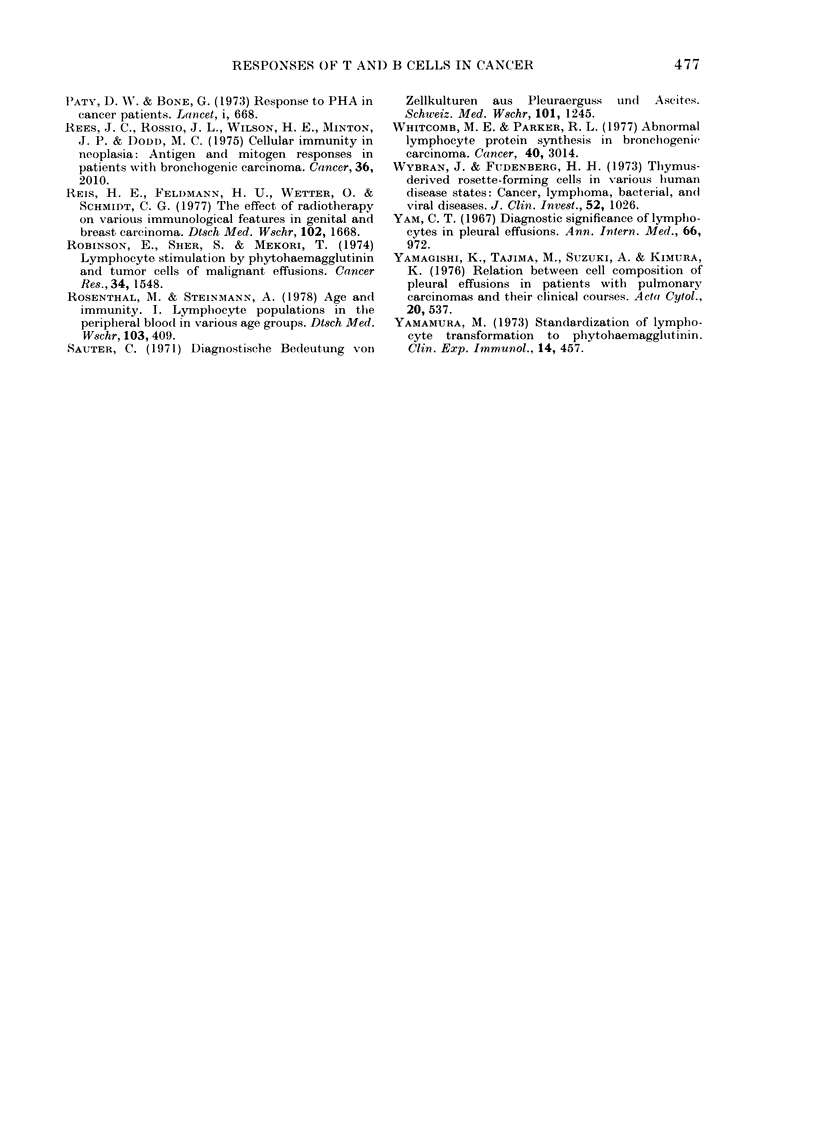# T- and B-cell responses in patients with malignant pleural effusions.

**DOI:** 10.1038/bjc.1981.69

**Published:** 1981-04

**Authors:** A. M. Potrykus, G. Steinmann, E. Stein, R. Mertelsmann

## Abstract

Lymphocytes of lymphocyte-rich pleural effusions and venous blood from 16 cancer patients, 7 patients with benign pleural effusions and blood from 23 normal blood donors, were examined for cytological features, rosette-forming capacity, immunofluorescent staining, and PHA-stimulated DNA synthesis. Total protein and immunoglobulin levels were also determined. Metastatic effusions revealed approximately 40% higher content of immunoglobulins G, A and M (P less than 0.002) as well as of total protein (P less than 0.005) than non-metastatic pleural effusions. However, the serum of the non-cancer patients contained approximately 50% higher level of Ig than in cancer patients (P less than 0.001). Whilst there was no significant difference in the relative T- or B-cell contents of pleural effusions between cancer and non-cancer patients (P greater than 0.05) spontaneous proliferation of lymphocytes was significantly increased (P greater than 0.01), which led to a lower PHA-stimulated transformation index in pleural effusions from cancer patients than in all other lymphocyte sources examined (P less than 0.001).


*Br. J. Cancer* (1981) **43**, 471

### T- AND B-CELL RESPONSES IN PATIENTS WITH MALIGNANT PLEURAL EFFUSIONS

A. M. POTRYKUS, G. STEINMANN*, E. STEIN AND R. MERTELSMANN†

*From the First Medical Clinic, University of Hamburg, Hamburg, West Germany*

Received 17 September 1980 Accepted 7 January 1981

**Summary.—Lymphocytes of lymphocyte-rich pleural effusions and venous blood from 16 cancer patients, 7 patients with benign pleural effusions and blood from 23 normal blood donors, were examined for cytological features, rosette-forming capacity, immunofluorescent staining, and PHA-stimulated DNA synthesis. Total protein and immunoglobulin levels were also determined. Metastatic effusions revealed ~40% higher content of immunoglobulins G, A and M ($P<0{\cdot}002$) as well as of total protein ($P<0{\cdot}005$) than non-metastatic pleural effusions. However, the serum of the non-cancer patients contained ~50% higher level of Ig than in cancer patients ($P<0{\cdot}001$). Whilst there was no significant difference in the relative T- or B-cell contents of pleural effusions between cancer and non-cancer patients ($P>0{\cdot}05$) spontaneous proliferation of lymphocytes was significantly increased ($P>0{\cdot}01$), which led to a lower PHA-stimulated transformation index in pleural effusions from cancer patients than in all other lymphocyte sources examined ($P<0{\cdot}001$).**

METASTATIC PLEURAL EFFUSIONS of patients suffering from cancer of different organs sometimes contain very high numbers of lymphocytes (Yam, 1967; Lopes Cardozo & Harting, 1972). Yamagishi *et al.* (1976) found in patients with bronchogenic carcinoma that good clinical conditions were associated with high percentages of lymphocytes, whereas a deterioration of the clinical status was paralleled by an increase of macrophages in the pleural effusions, suggesting that a high number of lymphocytes is able to limit the extent of the disease for a certain period. Apart from this observation, nothing is known about the immunological importance and function of lymphocytes in lymphocyte-rich pleural effusions. In these effusions, metastatic cells and lymphocytes appear to live under optimum conditions of metabolism and nutrition (Sauter, 1971). Since these cells are accessible and can be studied repeatedly, lymphocytes from metastatic pleural effusions appear to be a good natural model for the study of some aspects of the immunological defence against malignant cells.

For this investigation, we selected pleural effusions rich in lymphocytes. We used various methods to characterize lymphocytes immunologically, and compared lymphocytes from metastatic pleural effusions with those from "benign" pleural effusions, as well as with peripheral blood lymphocytes from both patient groups and from normal controls, in order to evaluate the lymphocyte responses characteristic of malignant effusions.

### MATERIALS AND METHODS

*Patients.*—Twenty-three adult patients with recently diagnosed pleural effusions were examined. Effusions had been prescreened by cytological examinations, and the numbers of lymphocytes had been determined in order to select effusions with $10^5$ or more lymphocytes per ml pleural fluid for the metastatic pleural effusions. The "cancer

Present addresses: *Pathologisches Institut der Universität Kiel, Hospitalstr. 42, D-2300 Kiel, West Germany and †Memorial Sloan-Kettering Cancer Center, 1275 York Avenue, New York, N.Y. 10021, U.S.A.

patients" (n = 16) had cancer of the lung or breast complicated by metastatic effusions to the pleural cavity. Four patients had been irradiated in previous years, and their reactions in the PHA-stimulation assay were not considered, in order to exclude possible long-term effects of radiation on the PHA response (Hoppe *et al.*, 1977). The "non-cancer patients" (n = 7) had congestive heart failure. Pleural effusions with at least $10^4$ lymphocytes per ml were selected for this group in order to get a reasonable number of non-metastatic pleural effusions for comparison. The pleural effusions from only 3 patients contained enough lymphocytes to measure the PHA response reliably after a $1\frac{1}{2}$-year period of sampling in our and neighbouring hospitals. None of the patients had received immunosuppressive chemotherapy.

Twenty-three healthy blood donors served as a control in the standardization of the methods. They ranged in age from 23 to 41 years, with a mean of 30 years. Males predominated (74%).

*Separation of lymphocytes and identification of cells.*—Blood was taken by routine venepuncture and anticoagulated with preservative-free heparin (20 u/ml). Patients with pleural effusions were asked to change their body positions at short intervals before puncture of the pleural cavity. Pleural fluid was also anticoagulated with preservative-free heparin (20 u/ml).

Mononuclear cells were separated from the peripheral blood on a Ficoll–Hypaque gradient using the method of Bøyum (1968). Cells of the pleural effusions were concentrated by centrifugation to a concentration of at least $10^9$ cells/l before separation. Separation was done by the same method. The cells were then washed ×3 and resuspended in RPMI 1640 medium. The whole procedure was carried out under sterile conditions.

*Identification of cells.*—Before and after separation of the lymphocytes, smears of venous blood and pleural effusions were prepared and stained panoptically with May-Grünwald–Giemsa. In order to identify mononuclear macrophages and monocytes, additional smears were prepared and cytochemically analysed for nonspecific esterase activity by means of a slight modification of the α-naphthyl acetate assay of Löffler (1961). Pleural effusions which yielded >1% macrophages or tumour cells after lymphocyte separation were excluded from the study.

*Rosette test.*—Mononuclear cells were suspended in RPMI 1640 containing 25mM Hepes buffer and 2% L-glutamine at a concentration of $2 \times 10^9$ cells/l. 0·2 ml washed sheep red blood cells (SRBC) were absorbed 1:1 with foetal calf serum (FCS) and added to 1 ml suspension of mononuclear cells. Cells were incubated for 5 min at 22°C, pelleted for 5 min at 60 *g*, and stored at 4°C overnight. After gentle resuspension, 0·02 ml was placed on a slide, 5 μl trypan blue was added, and this mixture was covered with a cover glass. Cells were examined with a Normarsky microscope and classified as: (1) rosette-forming cells (RFC) when at least 3 SRBC attached to their surface, (2) blue-stained non-vital lymphocytes, or (3) non-rosette-forming cells (non-RFC). Monocytes were excluded by nonspecific esterase staining (Aiuti *et al.*, 1975).

*Immunofluorescent staining.*—Mononuclear cells were resuspended in RPMI 1640 at a concentration of $10^{10}$ cells/l, kept for 30 min at 37°C, washed, and incubated for 30 min at 4°C with fluorescein-conjugated monospecific (IgG, IgA, or IgM) antisera from goat supplemented with 0·02% sodium azide. Cells were washed ×3 with PBS and the pellet resuspended in PBS–glycerol. Two drops were placed on a slide, covered, and examined with an epi-illuminated fluorescence microscope. Cells were classified as surface immunoglobulin-bearing cells (SIgBC) or non-SIgBC (Aiuti *et al.*, 1975; Horwitz & Lobo, 1975).

*PHA-stimulated DNA synthesis.*—Under sterile conditions, mononuclear cells were resuspended at a concentration of $10^9$ cells/l in RPMI 1640 supplemented with 15% FCS, 15mM Hepes buffer, 2% L-glutamine, 100 iu/ml penicillin, and 0·1 mg/ml streptomycin. Phytohaemagglutinin (PHA-P from DIFCO, Detroit, Michigan, U.S.A.) was dissolved in 5 ml bidistilled water and passed through a Millipore filter. Mononuclear cells were cultured in microtitre plates and supplemented with 0·025ml aliquots of a 0·025% dilution of PHA-P per ml cell suspension. Cells were incubated in a 5% $CO_2$–air environment at 37°C and 100% relative humidity for 72 h. Six hours before harvesting, 0·5 μCi $^3$H-thymidine was added to each culture. After a total of 78 h, cells were collected on fibreglass filters using an automated cell harvester. The rate of DNA synthesis was determined by measuring the [$^3$H]-dT uptake of the cells by liquid-scintillation counting. After correction of quenching, the results for each group of patients were expressed as the mean dissociations per minute (d/min) for stimulated and unstimulated cultures, or as the transformation index (TI) according to Yamamura (1973), which is the ratio of the d/min of stimulated cultures to the d/min of unstimulated cultures.

*Protein and immunoglobulin assays.*—Protein electrophoresis was carried out on agarose matrices with the standard method. Quantitative immunoelectrophoresis was performed by radioimmunodiffusion on standardized IgG, IgM and IgA plates (Behring, Marburg, Germany). A reference curve for each protein component was established using the standards supplied by the manufacturer. The concentrations of the various immunoglobulins were determined by comparing the developed precipitation rings with the calibrated reference curve.

*Statistical comparisons and normal values.*—The mean and standard deviation (s.d.) were determined for each group. Mean values were compared by means of the non-parametric U-test of Mann and Whitney, because this test does not require a Gaussian distribution of measured values, and is nearly as efficient as Student's $t$ test.

The healthy blood donors showed the following values (mean ± s.d.): lymphocytes $2{\cdot}34 \pm 0{\cdot}86 \times 10^9$/l; RFC 70·9 ± 7·5%; SIgBC 10·1 ± 3·0%; null cells 19·9 ± 2·2%; T-cell/B-cell ratio: 7·0 : 1; TI: 39·2 ± 6·6.

### RESULTS

Cancer patients had cytologically established metastases to the pleural cavity. The pleural effusions contained a variable number of malignant cells ($0{\cdot}02$–$50 \times 10^6$/l).

In the non-cancer patients, cancer was excluded by all available diagnostic methods, including a follow-up examination several months after analysis of their effusions. Their pleural effusions contained only benign cells.

The average value of lymphocytes in the venous blood (Table I) for the group of non-cancer patients is normal for this age group (Rosenthal & Steinmann, 1978). Cancer patients had a slightly, but significantly ($P < 0{\cdot}05$) lower average. A 9-fold higher mean lymphocyte count was seen in the pleural effusions of cancer patients ($P < 0{\cdot}001$) under the conditions of our selection.

### *Lymphocyte subpopulations*

The average number of T cells (RFC) in peripheral blood of cancer patients ($0{\cdot}905 \times 10^9$/l) was slightly lower than in non-cancer patients ($1{\cdot}161 \times 10^9$/l) but the difference was not significant ($P > 0{\cdot}05$). Both values lie in the normal range for this age group (Rosenthal & Steinmann, 1978).

While the absolute concentration of T cells in pleural effusions of cancer patients was considerably higher, there was no significant difference between non-cancer patients and cancer patients in the relative concentration of T cells ($P > 0{\cdot}2$).

TABLE I.—*Lymphocyte counts (mean × $10^{-9}$/l ± s.d.) of cancer patients and patients with other diseases*

| | n | M/F | Age (years) (mean ± s.d.) | Venous blood | Pleural effusion |
|---|---|---|---|---|---|
| Cancer patients | 16 | 7/9 | 64·4 ± 11·6 | 1·390 ± 0·871 | 0·841 ± 0·716 |
| Non-cancer patients | 7 | 4/3 | 60·3 ± 19·5 | 1·598 ± 0·333 | 0·097 ± 0·090 |

TABLE II.—*Lymphocyte subpopulations in pleural effusions (PE) and venous blood (VB) from cancer and non-cancer patients*

| Group | Source | No. of patients | T cells (%) | B cells (%) | Null cells (%) | T/B |
|---|---|---|---|---|---|---|
| Non-cancer | VB | 7 | 72·7 ± 8·4 | 20·9 ± 5·0 | 6·4 ± 6·1 | 3·48 |
| Cancer | PE | 16 | 70·3 ± 16·9 | 20·8 ± 13·4 | 8·9 ± 7·7 | 3·38 |
| Non-cancer | PE | 7 | 65·7 ± 18·6 | 24·0 ± 14·7 | 10·3 ± 6·3 | 2·74 |
| Cancer | VB | 16 | 65·1 ± 11·6 | 24·5 ± 8·1 | 10·4 ± 5·9 | 2·68 |

When the proportion of T cells in peripheral blood was compared with that in pleural effusions, there was no significant difference in the group of cancer patients ($P > 0{\cdot}05$) though the proportion was higher in pleural effusions. The effusions of non-cancer patients also revealed a slightly higher proportion of T cells than in their venous blood, but the difference was also not statistically significant ($P > 0{\cdot}2$) (Table II).

The B-cell (SIgBC) counts in peripheral blood of cancer patients (mean: $0{\cdot}334 \times 10^9$/l) did not differ ($P > 0{\cdot}2$) from those of non-cancer patients (mean: $0{\cdot}340 \times 10^9$/l). Both values, however, were higher than in young healthy persons ($P < 0{\cdot}01$). Especially was the T-cell/B-cell ratio found to be much lower (Table II).

Pleural effusions also showed about the same relative concentrations of B cells in cancer patients and non-cancer patients ($P > 0{\cdot}2$).

Cancer patients showed slightly lower B-cell proportions in pleural effusions than in venous blood ($P > 0{\cdot}05$). A similar difference in non-cancer patients was also not significant ($P > 0{\cdot}1$) (Table II).

We found a lower proportion of marker-silent cells (so-called "null" cells) (Non-RFC and Non-SIgBC) in the lymphocytes in the venous blood and pleural effusions of cancer and non-cancer patients than in young healthy persons ($P < 0{\cdot}01$). Venous blood of non-cancer patients especially contained few null cells (Table II).

*Response to PHA*

Lymphocytes from peripheral blood of cancer and non-cancer patients revealed lower transformation indices (TI) than in young healthy donors ($P < 0{\cdot}01$) but were indistinguishable from those of healthy persons of the same age group (Yamamura, 1973; Barnes *et al.*, 1975). Lymphocytes from pleural effusions from non-cancer patients showed even lower TIs ($P < 0{\cdot}001$).

The lymphocytes from the venous blood and the pleural effusions of cancer patients clearly showed higher rates of DNA synthesis in the unstimulated state than lymphocytes from both blood and effusions of non-cancer patients ($P < 0{\cdot}01$; Table III). Consequently, cancer patients' pleural effusions had the lowest TI of all the lymphocyte sources.

*Proteins and immunoglobulins*

Cancer patients showed higher levels of

TABLE III.—*PHA-stimulated DNA synthesis in cultured lymphocytes from pleural effusions and venous blood of cancer and non-cancer patients*

| Group | Source | No. of patients | [$^3$H]-dT uptake (d/min) (mean ± s.d.) Unstimulated | PHA stimulated | TI* |
|---|---|---|---|---|---|
| Non-cancer | VB | 3 | 744 ± 212 | 22115 ± 16263 | 29·7 |
| Cancer | VB | 12 | 2249 ± 2509 | 43030 ± 38321 | 19·1 |
| Non-cancer | PE | 3 | 995 ± 307 | 14432 ± 15314 | 15·4 |
| Cancer | PE | 12 | 2820 ± 2418 | 15847 ± 15839 | 5·6 |

$$* \text{TI} = \frac{\text{d/min PHA-stimulated}}{\text{d/min unstimulated}}$$

TABLE IV.—*Protein and immunoglobulin concentrations in serum and pleural effusions from cancer and non-cancer patients*

| Group | Source of protein | No. of patients | Total protein (mg/l) | Immunoglobulin (%) G+A+M | IgG | IgA | IgM |
|---|---|---|---|---|---|---|---|
| Cancer | Serum | 16 | 67·98 | 20·3 | 14·9 | 3·7 | 1·7 |
| Non-cancer | Serum | 7 | 64·25 | 31·1 | 24·6 | 4·6 | 1·9 |
| Cancer | Pleural effusion | 16 | 38·00 | 20·0 | 15·5 | 3·3 | 1·2 |
| Non-cancer | Pleural effusion | 7 | 23·06 | 14·6 | 11·6 | 2·2 | 0·8 |

total protein ($P < 0{\cdot}005$) and of IgG, IgA, and IgM ($P < 0{\cdot}002$) in their pleural effusions than non-cancer patients and lower Ig levels in their sera than in non-cancer patients ($P < 0{\cdot}05$) (Table IV).

### DISCUSSION

Lymphocyte-rich pleural effusions occur in $\sim 15\%$ of cancer patients whose cancer has spread to this organ (Steinmann, unpublished). In patients in whom a pleural effusion has been acquired for a reason other than cancer, lymphocyte-rich pleural effusions are only occasional. This fact leads to the hypothesis that the malignant cells themselves were responsible for the gathering of lymphocytes, and also that they attracted certain subpopulations (Djeu *et al.*, 1976).

In metastatic pleural effusions from cancer patients, the distribution of B, T, and null lymphocytes was found to be nearly identical to that in the group of patients with benign diseases complicated by pleural effusions. There was a slight but not significant tendency for T cells to occur in higher numbers in metastatic pleural effusions. This does not confirm the report of Djeu *et al.* (1976) that the lymphocytes in metastatic pleural effusions were predominantly T cells. This discrepancy might be due to the use of a different RFC assay or to contamination by macrophages or tumour cells in the effusions examined by these authors.

In venous blood of cancer patients, we found a decreased percentage of RFC, which might correspond to a lower T-cell-mediated immune competence than in patients with benign diseases and healthy persons in the same age group. This observation agrees with previous reports (Wybran & Fudenberg, 1973; Oldham *et al.*, 1976). In contrast, there were no significant differences between cancer and non-cancer patients in the number of B cells in venous blood and pleural effusions. This agrees with the observation that cancer patients show no detectable evidence of decreased immunoglobulin synthesis (Aizawa & Southam, 1960).

The peripheral B-cell counts of both cancer patients and non-cancer patients were slightly higher than in younger healthy volunteers, probably due to the greater age.

We found higher levels of proteins and immunoglobulins in metastatic pleural effusions of cancer patients than in non-cancer patients, which did not parallel the B-cell counts in these effusions, suggesting that the Igs in the effusions of cancer patients were not only produced by the B cells in these effusions. The lower level of Ig in the serum of the cancer patients with pleural effusions than in non-cancer patients also supports the hypothesis of an intensive transport of Ig from the serum to the pleural effusion. It would be of interest to analyse why these antibodies are so ineffective in the defence of malignant cells (Mitchison, 1977).

PHA-stimulated DNA synthesis in blood lymphocytes of cancer patients and other patients has already been investigated by numerous research groups (Barnes *et al.*, 1975; Han & Takita, 1972; Hoppe *et al.*, 1977; Pary & Bone, 1973; Rees *et al.*, 1975; Reis *et al.*, 1977; Robinson *et al.*, 1974; Whitcomb & Parker, 1977). Most of the authors found that PHA-stimulated DNA synthesis in blood lymphocytes of patients with early-stage cancer was reduced slightly if at all. A clear reduction in DNA synthesis was not found until preterminal stages and after radiation therapy. We also did not observe a significant difference in DNA synthesis by blood lymphocytes between cancer patients and non-cancer patients. On the other hand, we found a significantly increased rate of spontaneous DNA synthesis in the lymphocytes of both peripheral blood and pleural effusions from cancer patients.

Several years ago, Lopes Cardozo & Harting (1972) described the PHA-induced blast transformation of lymphocytes from metastatic pleural effusions. In contrast to our study, they used only morphological criteria of blast transformation, which they reported was generally good, even when the patients had been treated with chemotherapeutic agents.

In our study, the lymphocytes from malignant pleural effusions showed a distinctly smaller increase in the rate of DNA synthesis in response to PHA than lymphocytes from benign pleural effusions. This difference was even greater when the lymphocytes of malignant pleural effusions were compared with blood lymphocytes. The transformation index (TI) of lymphocytes of malignant pleural effusions was 30% of the TI of lymphocytes from benign pleural effusions, 25% of the TI of blood lymphocytes from the same cancer patients, and 16% of the TI of blood lymphocytes from non-cancer patients.

The low TI of the lymphocytes from metastatic pleural effusions in our studies was influenced by the relatively high rate of spontaneous DNA synthesis. Since T lymphocytes respond to stimulation by allogeneic antigens (Clot *et al.*, 1975) stimulation by tumour antigens or by other antigens in cancer-associated diseases might be responsible for the high rate of spontaneous DNA synthesis, since the rate of spontaneous synthesis by blood lymphocytes from cancer patients was also high. This hypothesis is supported by findings of Robinson *et al.* (1974), who studied the stimulation of blood lymphocytes by tumour cells from pleural effusions, and found a relatively low degree of additional stimulation by PHA.

The rate of PHA-stimulated DNA synthesis in lymphocytes from benign pleural effusions was also relatively low, suggesting additional cancer independent factors suppressing the PHA response in pleural-effusion lymphocytes. Robinson *et al.* (1974) considered the possibility of a factor in pleural effusions that could inhibit the stimulation of lymphocytes. This factor was speculative, but might be similar to the one in mouse ascites, which prolongs the survival of allografts (Biran *et al.*, 1969).

While our immunological characterization of lymphocytes in lymphocyte-rich pleural effusions of cancer and non-cancer patients did not show any major differences in relative B- and T-cell contents, the analysis of the more functional parameters spontaneous DNA synthesis, PHA-stimulated DNA synthesis, and Ig content revealed an enhanced activity of lymphocytes of both T and B lineage in metastatic pleural effusions.

### REFERENCES

AIUTI, F., CEROTTINI, J. C., COOMBS, R. R. A. & 17 others (1975) Identification, enumeration, and isolation of B and T lymphocytes from human peripheral blood. *Clin. Immunol. Immunopathol.*, **3**, 584.

AIZAWA, M. & SOUTHAM, C. M. (1960) Serum antibodies following homo-transplantation of human cancer cells. *Ann. N.Y. Acad. Sci.*, **87**, 293.

BARNES, E. W., FARMER, A., PENHALE, W. J., IRVINE, W. J., ROSCOE, P. & HORNE, N. W. (1975) Phytohemagglutinin-induced lymphocyte transformation in newly presenting patients with primary carcinoma of the lung. *Cancer*, **36**, 187.

BIRAN, S., BEN HUR, N. & ROBINSON, E. (1969) Studies on the behavior of skin autografts following their *in vitro* treatment with cancer cells. *Israel J. Med. Sci.*, **5**, 1977.

BØYUM, A. (1968) Isolation of mononuclear cells and granulocytes from human blood. *Scand. J. Clin. Lab. Invest.*, **21** (Suppl. 97), 77.

CLOT, J., MASSIP, H. & MATHIEU, O. (1975) *In vitro* studies on human B and T cell purified populations; Stimulation by mitogens and allogenic cells and quantiative binding of phytomitogens. *Immunology*, **29**, 445.

DJEU, J. Y., MCCOY, J. L., CANNON, G. B., REEVES, W. J., WEST, W. H. & HERBERMAN, R. B. (1976) Lymphocytes forming rosettes with sheep erythrocytes in metastatic pleural effusions. *J. Natl Cancer Inst.*, **56**, 1051.

HAN, T. & TAKITA, H. (1972) Immunologic impairment in bronchogenic carcinoma: A study of lymphocyte response to phytohemagglutinin. *Cancer*, **30**, 616.

HOPPE, R. T., FUKS, Z. V., STROBER, S. & KAPLAN, H. S. (1977) The long term effects of radiation on T and B lymphocytes in the peripheral blood after regional irradiation. *Cancer*, **40**, 2071.

HORWITZ, D. A. & LOBO, P. I. (1975) Characterization of two populations of human lymphocytes bearing easily detectable surface immunoglobulin. *J. Clin. Invest.*, **56**, 1464.

LÖFFLER, H. (1961) Cytochemischer Nachweis von unspezifischer Esterase in Ausstrichen. *Klin. Wschr*, **39**, 1220.

LOPES CARDOZO, E. & HARTING, M. C. (1972) On the function of lymphocytes in malignant effusions. *Acta Cytol.*, **16**, 307.

MITCHISON, N. A. (1977) T and B Cells in Cancer. *B and T Cells in Immune Recognition*. Eds Loor & Roelants. London: John Wiley & Sons. p. 337.

OLDHAM, R. K., WEESE, J. L., HERBERMAN, R. B. & 12 others (1976) Immunological monitoring and immunotherapy in carcinoma of the lung. *Int. J. Cancer*, **18**, 739.

PATY, D. W. & BONE, G. (1973) Response to PHA in cancer patients. *Lancet*, i, 668.

REES, J. C., ROSSIO, J. L., WILSON, H. E., MINTON, J. P. & DODD, M. C. (1975) Cellular immunity in neoplasia: Antigen and mitogen responses in patients with bronchogenic carcinoma. *Cancer*, **36**, 2010.

REIS, H. E., FELDMANN, H. U., WETTER, O. & SCHMIDT, C. G. (1977) The effect of radiotherapy on various immunological features in genital and breast carcinoma. *Dtsch Med. Wschr*, **102**, 1668.

ROBINSON, E., SHER, S. & MEKORI, T. (1974) Lymphocyte stimulation by phytohaemagglutinin and tumor cells of malignant effusions. *Cancer Res.*, **34**, 1548.

ROSENTHAL, M. & STEINMANN, A. (1978) Age and immunity. I. Lymphocyte populations in the peripheral blood in various age groups. *Dtsch Med. Wschr*, **103**, 409.

SAUTER, C. (1971) Diagnostische Bedeutung von Zellkulturen aus Pleuraerguss und Ascites. *Schweiz. Med. Wschr*, **101**, 1245.

WHITCOMB, M. E. & PARKER, R. L. (1977) Abnormal lymphocyte protein synthesis in bronchogenic carcinoma. *Cancer*, **40**, 3014.

WYBRAN, J. & FUDENBERG, H. H. (1973) Thymus-derived rosette-forming cells in various human disease states: Cancer, lymphoma, bacterial, and viral diseases. *J. Clin. Invest.*, **52**, 1026.

YAM, C. T. (1967) Diagnostic significance of lymphocytes in pleural effusions. *Ann. Intern. Med.*, **66**, 972.

YAMAGISHI, K., TAJIMA, M., SUZUKI, A. & KIMURA, K. (1976) Relation between cell composition of pleural effusions in patients with pulmonary carcinomas and their clinical courses. *Acta Cytol.*, **20**, 537.

YAMAMURA, M. (1973) Standardization of lymphocyte transformation to phytohaemagglutinin. *Clin. Exp. Immunol.*, **14**, 457.